# Mutation of NDUFAF2 Linked to Mitochondrial Complex I Deficiency

**DOI:** 10.7759/cureus.86670

**Published:** 2025-06-24

**Authors:** Anwar R Alhamad, Aziza Mushiba, Huda Alkhawaja, AbdullabAli PeerZada, Shahad Bawazeer, Mohammed Saleh

**Affiliations:** 1 Genetics, Maternity and Children's Hospital in Al-Ahsa, Hofuf, SAU; 2 Pediatrics, King Fahad Medical City, Riyadh, SAU; 3 Pediatrics, Maternity and Children's Hospital in Al-Ahsa, Hofuf, SAU; 4 Laboratory Medicine, King Fahad Medical City, Riyadh, SAU; 5 Genetics, King Fahad Medical City, Riyadh, SAU

**Keywords:** case report, episodic respiratory failure, mitochondrial complex i deficiency, ndufaf2, recurrent encephalopathy

## Abstract

Mitochondrial complex I deficiency is an autosomal recessive disorder caused by homozygous mutations in the reduced form of nicotinamide adenine dinucleotide (NADH). It is characterized by a wide range of signs and symptoms that affect numerous human systems and organs. This disease causes neurological issues, including encephalopathy, recurrent epilepsy, intellectual disability, ataxia, and involuntary movements. The initial step of the mitochondrial respiratory chain, during which protons are transported across the inner mitochondrial membrane along with electron transfer from NADH to ubiquinone, is catalyzed by NADH: ubiquinone oxidoreductase. In this case report, we describe a patient presenting with severe, rapidly progressive neurological loss who harbored a novel mutation in *NDUFAF2* identified using exome sequencing. At six  months of age, her mother noticed delayed motor development. Thereafter, the patient developed metabolic acidosis and abnormal movements, mimicking seizures triggered by aspiration pneumonia, with elevated serum lactate levels. Genetic testing revealed a c.127G>A mutation in *NDUFAF2*, consistent with mitochondrial complex I deficiency. This case highlights the utility of exome sequencing as a powerful and cost-effective tool for diagnosing clinically heterogeneous disorders such as mitochondrial diseases. Mitochondrial complex I deficiency is an important differential diagnosis in patients with recurrent central hypoventilation. Our findings expand the mutational spectrum of this rare disease.

## Introduction

Mitochondria contain groups of proteins that transport electrons through four chain reactions (complexes I-IV) to produce energy for cellular processes. Mitochondrial complex I deficiency leads to the onset of the most common defect in the oxidative phosphorylation system during childhood. Mutations in the nuclear- or mitochondria-encoded structural subunits of the enzyme or any of the rapidly growing numbers of nuclear-encoded complex I assembly factors can cause inherited complex I deficiency [1]. This disease exhibits wide clinical heterogeneity, ranging from neonatal-onset lactic acidosis to Leigh disease and other encephalomyopathies [1].

Nuclear type 10 mitochondrial complex I deficiency is an autosomal recessive disease caused by a mutation in the NADH ubiquinone oxidoreductase complex assembly factor 2 (*NDUFAF2*) gene located on chromosome 5q12 [2]. The mutation manifests clinically as muscle soreness and hypotonia [3]. Nine assembly factors, *NDUFAF1, NDUFAF2, NDUFAF3, NDUFAF4, C20ORF7, C8ORF38, NUBPL, FOXRED1,* and *ACAD9,* are associated with human diseases [1]. Leigh syndrome is associated with *NDUFAF2, C8ORF38, C20ORF7,* and *FOXRED1 *[1].

According to a previous study, most gene mutations in patients with mitochondrial complex I deficiency (24 cases) are in *NDUFS4* (21 cases); however, *NDUFV1* and *NDUFS2* mutations also frequently occur (14 and 12 cases, respectively) [1]. *NDUFAF2* is highly expressed in esophageal squamous cell carcinoma as well as in the heart, skeletal muscle, liver, and fibroblasts [4]. *NDUFAF2 *protein functions as a molecular chaperone for the assembly of mitochondrial complex I, which transfers electrons from NADH to the respiratory chain. Ubiquinone is thought to be the immediate electron acceptor of this enzyme [5]. Mitochondrial complex I deficiency (OMIM#618233) is characterized by apnea, episodic respiratory failure, poor feeding, episodic encephalopathy, global developmental delay, ataxia, leukoencephalopathy, and mitochondrial complex I deficiency in various tissues.

Comprehensive evaluation of mitochondrial complex I deficiency showed that 82 of 172 individuals with nuclear-encoded complex I deficiency had detailed findings on brain magnetic resonance imaging. Only 13% of the examined patients had isolated basal ganglia lesions, 28% had isolated brainstem lesions, and 24% had lesions in both the basal ganglia and brainstem [1].

The clinical course and prognosis of patients with mitochondrial complex I deficiency vary widely and may depend on specific genetic defects, age of onset, organ involvement, and other factors [6]. Here, we report the clinical, biochemical, and molecular data of a nine-month-old girl who presented with a typical course of Leigh disease and required intubation at the age of six months. Exome sequencing revealed a previously unreported mutation in *NDUFAF2.* The cousins of this patient exhibited similar clinical presentations.

## Case presentation

Patient information

A nine-month-old Saudi infant girl born full-term via cesarean section because of breech presentation was admitted to the neonatal intensive care unit for three days because of poor feeding and decreased activity. The infant was discharged against medical advice. The infant’s behavior was normal, except for excessive crying, vertical nystagmus, not visually following an object presented to her, and mild hypotonia until the age of six months, when she developed fever and recurrent apnea. She was admitted to the pediatric intensive care unit and diagnosed with lactic acidosis. Her oxygen saturation level decreased to 80%, and respiratory failure type II secondary to aspiration bronchopneumonia was observed. Her condition progressively worsened because of an abnormal tonic-clonic movement in the upper limbs for a few seconds, which led to the need for ventilation support on three occasions. During hospitalization, she had high blood pressure, reaching 131/70 mmHg, and was treated with an antihypertensive drug (hydralazine).

The parents were first cousins; additionally, two children from maternal side with same phenotype died at 18 months and one year of age. However, no genetic testing was performed.

Clinical findings

The infant was visibly unwell, on mechanical ventilation under sedation, was not dysmorphic, and showed bilateral nystagmus. Laboratory tests revealed increased blood lactate and lactate dehydrogenase levels and respiratory acidosis (Table 1).

**Table 1:** Blood investigation results including blood gas which shows respiratory acidosis but the others, complete blood count, renal, liver function and biochemistry all within normal

Laboratory test	Patient value	Reference value
Venous blood gas		
pH	7.12	7.35-7.45
Partial pressure of CO2	99	35-45 mmHg
Bicarbonate	24	22-28 mmol/L
Lactate	3.5	0.5-2.2 mmol/L
Complete blood count		
White blood cells	8.7	10^3^/ulu
Hemoglobin	12	g/dl
Platelets	238	10^3^/uL
Renal functions		
Blood urea nitrogen	3.2	1.7-8.30 mmol/L
Creatinine	35	49-115 umol/L
Liver functions		
Aspartate aminotransferase	38	15-37 U/L
Alanine aminotransferase	19	46-116 U/L
Electrolytes		
Sodium	140	135-145 mmol/L
Potassium	4.2	3.5-4.5 mmol/L
Calcium	2.43	2.12-2.52 mmol/L
Chloride	100	98-115 mmol/L
Magnesium	0.84	0.74-0.99 mmol/L
Phosphorus	2.6	0.8-1.5 mmol/L
Biochemistry		
Alkaline phosphatase	164	46-116 U/L
Lactate dehydrogenase	847	100-190 U/L
Uric acid	363.95	155-428 Umol/L

Blood, urine, and tracheal aspirate cultures initially tested negative; however, repeated blood cultures tested positive for *Staphylococcus epidermidis*, and a tracheal aspirate sample tested positive for *Pseudomonas aeruginosa*. 

Differential diagnosis of episodic respiratory failure should consider central nervous system causes, neuromuscular disorders, lung and metabolic disease.

**Figure 1:**
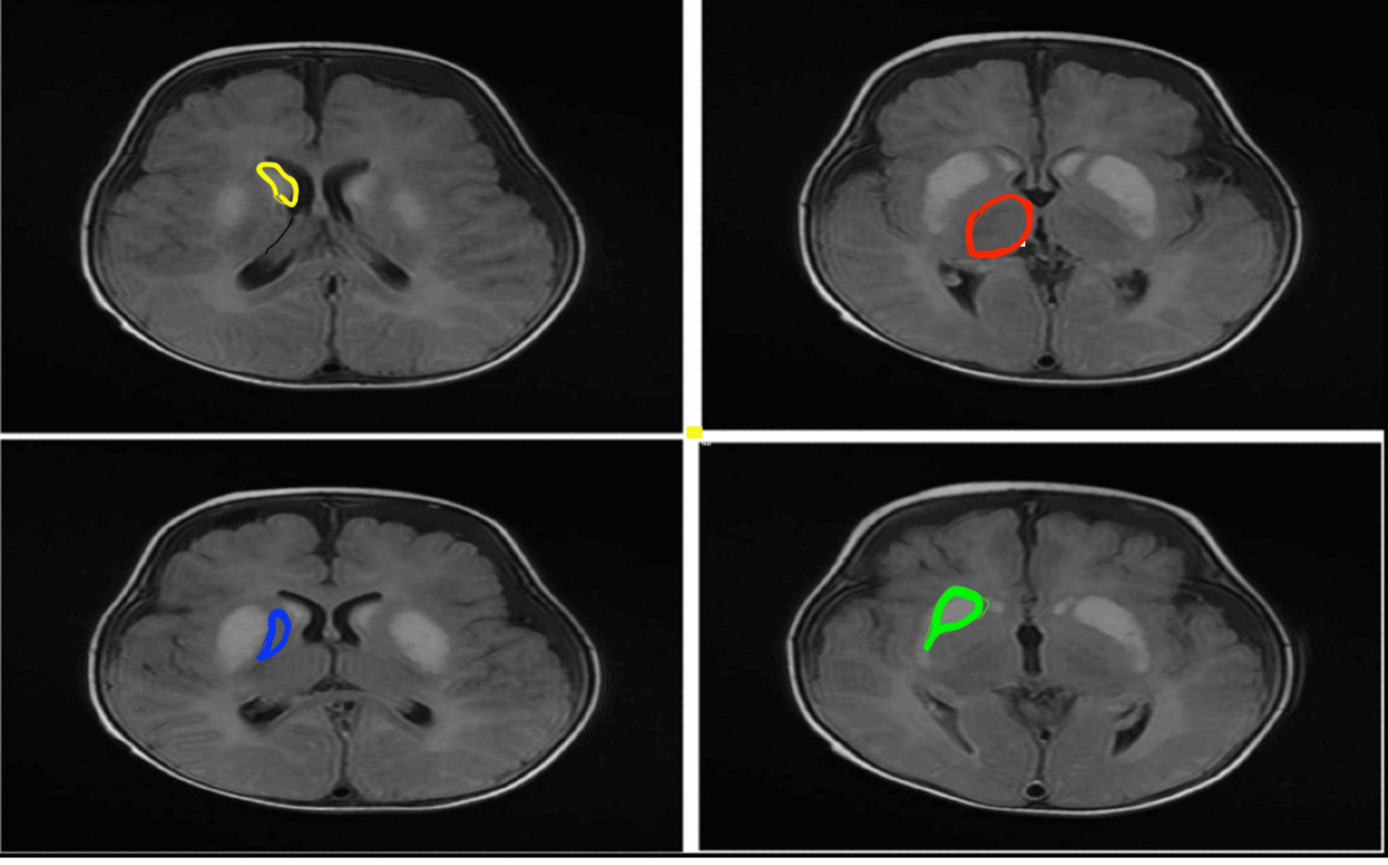
Brain MRI demonstrates basal ganglia (a) Caudate nucleus (yellow); (b) thalamus (red); (c) globus pallidus (blue); (e) putamen (green)

Brain magnetic resonance imaging (Figure 1) revealed bilateral, symmetrical, and abnormal signal intensities in the caudate, putamen, mesial temporal cortex, central tegmental tract, and cerebellar nuclei. High signal intensity was evident on T2- and fluid-attenuated inversion recovery-weighted images with diffusion restriction. Bilateral frontal subdural cerebrospinal fluid collection was also observed. The maximum thickness noted on the left and right sides measured 0.5 and 0.2 cm, respectively. The optic chiasm showed an abnormally high central signal intensity on T2-weighted images and low signal intensity on fluid-attenuated inversion recovery images. Effusion was observed bilaterally in the mastoid air cells and middle ear cavity, suggesting metabolic mitochondrial diseases such as Leigh and Leigh-like syndrome.

Recurrent respiratory acidosis, high lactate, and abnormal signal intensities in the basal ganglia raise suspicion of mitochondrial disease.

Clinical intervention

Mitochondrial disease was suspected based on the clinical, laboratory, and imaging findings. Accordingly, biotin (10 mg/kg/day) and thiamine (40 mg/kg/day) were administered, considering the treatable nature of biotin-thiamine-responsive basal ganglia diseases. However, the patient’s condition did not improve.

DNA samples were collected and sent to a tertiary hospital for genetic diagnosis (in-house laboratory analysis was not available). The patient experienced recurrent episodes of central hypoventilation with encephalopathy and required prolonged intubation support until she developed refractory hypoxemia, which required high-frequency oscillatory ventilation, followed by hypotension and multiorgan failure, which resulted in her death.

Diagnosis 

**Figure 2:**
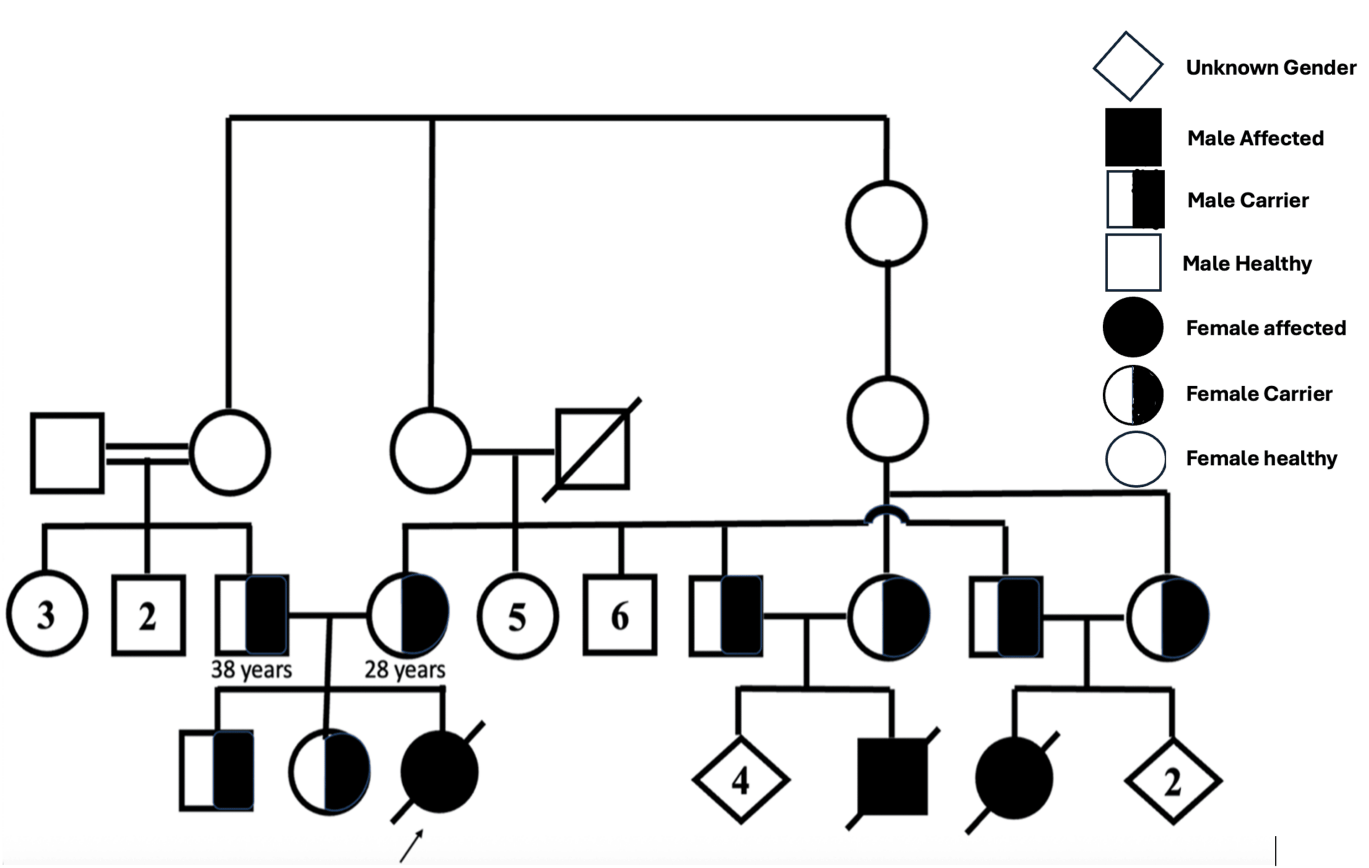
Extended family pedigree showing consanguineous marriage with the arrow pointing to the affected case (solid box) and other carrier siblings (half solid box) Generated with Microsoft PowerPoint for Mac, Version 16.98 (Microsoft Corp., Redmond, WA, USA)

Whole-exome sequencing led to the identification of a homozygous variant of uncertain significance in *NDUFAF2* (NM_174889.4: c.127G>A:p. Gly43Arg). Pathogenic variants of this gene are associated with autosomal recessive mitochondrial complex deficiency, nuclear type 10. Segregation analysis revealed that both parents carried the same mutation (Figure 2). Two cousins of the deceased patient, who were born to two maternal uncles and their wives with carrier status, died under similar conditions. 

The family was counseled about the disease, its recurrence risk, and preventive measures through prenatal diagnosis or prenatal gestation diagnosis. Given the lack of effective treatment for this disease, the prognosis is generally poor; only supportive care can be offered, and prenatal diagnosis or prenatal gestation diagnosis is advised.

Result

Missense variants in *NDUFAF2* correspond to the phenotype of the gene mutation in the patient, and segregation analysis of both parents revealed that they carried the same gene mutation. Two similar cases with the same clinical presentation died in the same family (son and daughter of mother's brothers) without diagnosis or genetic study, in silico parameters, and allele frequency of the mutation in gnomAD. Our data support this change as the cause of the disease, and the variant was upgraded to a likely pathogenic variant.

**Figure 3:**
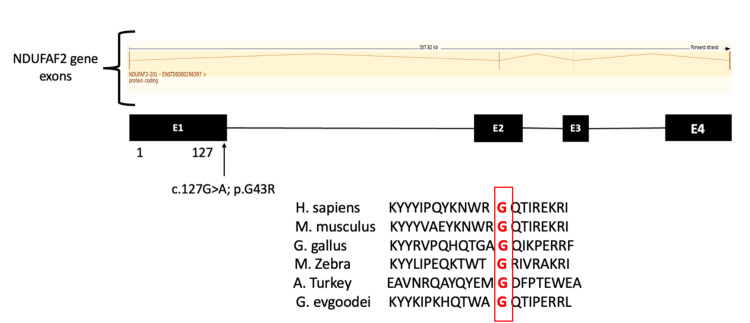
Exon map of NDUFAF2 variant and conservation of mutated nucleotides across species

The *NDUFAF2* variant is conserved across species (Figure 3), suggesting its deleterious nature and contribution to the phenotype observed in the present case. 

**Figure 4:**
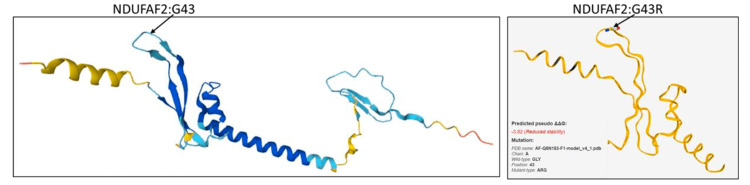
Difference between normal and abnormal (reduced stability) of structure prediction for NDUFAF2 Created using AlfaFold2 (UniProt databases) (DeepMind Technologies Ltd, London, United Kingdom) and Site-Directed Mutator software (Department of Biochemistry, University of Cambridge, Cambridge, United Kingdom)

These newly discovered links between genotypes and phenotypes are crucial, as they can accelerate diagnostic procedures. The majority of complex I deficiency events remain incurable; hence, palliative care is provided to most patients [3]. To understand the pathogenicity of this mutation, its expression level should be determined and compared with that in normal individuals (Figure 4).

## Discussion

Mitochondrial disease, characterized by mitochondrial complex I deficiency, is the most prevalent hereditary neurometabolic disease in children. Diagnosis of mitochondrial disease is challenging because it does not present specific clinical features. Specific clinical and laboratory tests, as well as a wider repertoire of genetic variations related to mitochondrial diseases, are prerequisites for enabling early diagnosis and improving quality of life.

Three previously reported cases harbored a homozygous nonsense mutation in *NDUFAF2* [7-9], and the fourth case harbored a missense A-to-T transversion, resulting in a methionine 1-to-leucine substitution with the same manifestation and outcomes as in our case, but without high lactic acid levels and preserved globus pallidus and thalami [8].

Neuroimaging alterations along with hyperlactatemia reinforced the suspicion of mitochondrial disease, warranting genetic investigation. Genomic DNA was enzymatically fragmented, and the target regions were enriched. These regions span approximately 41 Mb of the human coding exome (targeting >98% of the coding RefSeq from the human genome browser sequence GRCh37/hg19) and mitochondrial genome. The generated library was sequenced on an Illumina platform to obtain at least 20x coverage depth for >98% of the targeted bases. An in-house bioinformatics pipeline, including read alignment to the GRCh37/hg19 genome assembly, revised Cambridge Reference Sequence with a minor allele frequency of <1% in the gnomAD database, and disease-causing variants reported in Human Gene Mutation Database, ClinVar, or CentoMD were evaluated. The search for relevant variants focused on the encoding exons and the flanking ±10 intronic nucleotides of genes with clear gene-phenotype evidence. All potential inheritance patterns were considered. Family history and clinical information were used to evaluate the identified variants with respect to their pathogenicity and causality. Variants were classified according to American College of Medical Genetics guidelines [10]. All relevant variants related to the clinical phenotypes of patients were flagged.

The *NDUFAF2* variant c.127G>A p. Gly43Arg causes an amino acid change from glycine to arginine at position 43. This substitution occurred near a highly conserved donor splice site. It is classified as a variant of uncertain significance (grade 3) according to American College of Medical Genetics recommendations. Pathogenic variants of *NDUFAF2* are associated with mitochondrial complex I deficiency and nuclear type 10, characterized by episodic respiratory failure and encephalopathy, which matched the characteristics of our patient.

To the best of our knowledge, no prior publication has been identified reporting this variant. Two computational prediction tools for this variant, the Database Splicing Consensus Single Nucleotide Variant and the MaxEntScan engine, unanimously support a deleterious effect on the gene, but there is a need for further functional studies to fully confirm pathogenicity.

So we report this case to support any physician encountering a patient with the same variant and expand the mutated genes that cause the disease.

## Conclusions

In summary, a new mutation in NDUFAF2 (c.127G>A) caused mitochondrial complex I deficiency in our patient. These findings highlight the importance of an early diagnosis to prevent the occurrence of this disease in future generations of afflicted families.
